# Antibacterial Derivatives of Ciprofloxacin to Inhibit Growth of Necrotizing Fasciitis Associated Penicillin Resistant *Escherichia coli*

**DOI:** 10.1155/2013/517638

**Published:** 2013-05-02

**Authors:** Ronald Bartzatt, Suat L. G. Cirillo, Jeffrey D. Cirillo

**Affiliations:** ^1^University of Nebraska, College of Arts & Sciences, Durham Science Center, Department of Chemistry, Omaha, NE 68182, USA; ^2^Texas A & M Health Science Center, Department of Microbial and Molecular Pathogenesis, Bryan, TX 77807, USA

## Abstract

*Escherichia coli* (*E. coli*) is associated with necrotizing fasciitis (type I) and can induce enough damage to tissue causing hypoxia. Three ester derivatives of the broad-spectrum antibiotic ciprofloxacin were placed into bacteria culture simultaneously with the parent ciprofloxacin (drug 1) to ascertain the level of antibacterial activity. The n-propyl (drug 2), n-pentyl (drug 3), and n-octyl (drug 4) esters of ciprofloxacin were synthesized under mixed phase conditions and by microwave excitation. The formation of ester derivatives of ciprofloxacin modified important molecular properties such as Log *P* and polar surface area which improves tissue penetration, yet preserved strong antibacterial activity. The Log *P* values for drugs 1, 2, 3, and 4 became −0.701, 0.437, 1.50, and 3.02, respectively. The polar surface areas for drugs 1, 2, 3, and 4 were determined to be 74.6 Angstroms^2^, 63.6 Angstroms^2^, 63.6 Angstroms^2^, and 63.6 Angstroms^2^, respectively. These values of Log *P* and polar surface area improved tissue penetration, as indicated by the determination of dermal permeability coefficient (*K*_*p*_) and subsequently into the superficial fascial layer. All drugs induced greater than 60% bacterial cell death at concentrations less than 1.0 micrograms/milliliter. The ester derivatives of ciprofloxacin showed strong antibacterial activity toward penicillin resistant *E. coli*.

## 1. Introduction

Necrotizing fasciitis is an often fatal infection of the softtissue that will commonly begin after some form of trauma [1]. This softtissue infection involves the superficial fascial layers (or hypodermis) of the abdomen, extremities, or perineum [1]. This infection of the deep layers of skin and subcutaneous tissues easily spreads across the fascial plane. A polymicrobial infection is more common, involving gram-positive, gram-negative (i.e., *Escherichia col* (*E. coli*)), aerobic, and anaerobic bacteria [1]. Quick diagnosis, application of broad-spectrum antibiotics, and/or surgical intervention is required for successful patient outcome [1]. 

The three types of necrotizing fasciitis are based on (1) anatomy; (2) the depth of infection; and/or (3) the microbial source for the infection [2]. Type I infection is by polymicrobial incidents involving gram-positive cocci, Gram-negative rods, and anaerobes [2]. For Type I episodes, one type of bacteria can aid the survival and growth of another bacteria (this is synergy). Common type I category bacteria include *E. coli*, *Klebsiella*, *Staphylococcus aureus*, and *Streptococcus* species [2]. The Treatment of the infection requires strong broad-spectrum antibiotics administered intravenously. Type II necrotizing fasciitis is monomicrobial with rapidly progressing necrosis of multiple tissue layers that involves largely group A streptococcal bacteria [3]. Type III (gas gangrene) is mostly associated with *Clostridium perfringens* [3]. The presence of *E. coli* can induce necrotizing fasciitis with the excretion of a necrotic toxin (cytotoxic necrotizing factor 1) [4, 5]. Philippine studies showed that the most frequent isolates of necrotizing fasciitis included *E. coli* (44%), *Acinetobactor baumanni* (19%), *Staphylococcus aureus* (15%), and *Enterococcus faecium* (15%) [6]. *E. coli* was the most common bacteria associated with necrotizing fasciitis in Peshawar [7] and New York [8]. 

 Previous studies have shown clearly that molecular descriptors such as Log *P* and polar surface area are highly useful for estimating drug likeness of new drug candidates [9]. In addition, results of further investigations show that evaluating polar surface area is a highly efficient method of predicting oral absorption, intestinal absorption, membrane permeation, and drug transport properties [10, 11].

Clearly *E. coli* is associated with fatal incidents of necrotizing fasciitis. This study demonstrates that the modification of structural substituents of the broad-spectrum antibacterial agent ciprofloxacin will result in antibacterial agents having molecular properties beneficial for inhibiting the proliferation of *E. coli* and the clinical treatment of necrotizing fasciitis type infections. 

## 2. Materials and Methods

### 2.1. Reagents and Supplies

 Chemicals and reagents were analytical grade and obtained from Aldrich Chemical Company (P.O. Box 2060, Milwaukee, WI USA). All glassware utilized in synthesis was washed thoroughly in distilled water and baked to dryness. 

### 2.2. Molecular Modeling Software

 Molecular modeling and molecular properties were determined by utilizing ChemSketch v. 5 (90 Adelaide Street West, Toronto, ON, M5H 3V9, Canada) and Molinspiration (Liscie udolie 2, SK-841 04 Bratislava, Slovakia). The Dermal Permeability coefficient program (DERMWIN) estimates the dermal permeability coefficient (*K*_*p*_) and the dermally absorbed dose per event of these organic compounds (DERWIN v. 1.42, copyright 2000 U.S. Environmental Protection Agency). Statistical analysis was accomplished utilizing Microsoft EXCEL v. 14.0.6123.5001 (©2010 Microsoft Corporation). Values of Log *D* were determined by SPARC online calculator of properties (http://archemcalc.com/sparc/logKd/logkd.cfm).

### 2.3. Synthesis of Derivatives

For each of drugs 2, 3, and 4 derivatives of ciprofloxacin, a mass of 90 milligrams of ciprofloxacin was measured out into a large test tube. Determining the amount of ciprofloxacin to be 1.81*E* − 04 moles then add an equal number of moles of thionyl chloride (SOCl_2_, formula weight = 118.9 grams/mole, density = 1.638 grams/mL), with grams thionyl chloride determined by its density. The thionyl chloride is placed first in a dry test tube followed by the addition of solid ciprofloxacin. The thionyl chloride reacts with the carboxyl group found within the structure of ciprofloxacin.

The mixture is then subjected to microwave excitation for a total of three to five minutes which heats the mixture combination and must be monitored (due to fluctuations in energy of microwave instrument the mixture must be monitored for excess heat that induces degradation of the reagents). The reactant mixture is allowed to cool and 0.3 mL of 1-propanol (for drug 2), 1-pentanol (for drug 3), and 1-octanol (for drug 4) are added quickly. The formation of the ester reaction is immediate with excess alcohol or amine being removed by evaporation using vacuum pump drying. The ester derivatives are confirmed by infrared spectroscopy at 1735 cm^−1^ and stretch at 1760–1670 cm^−1^ and amides detected at 1630–1695 cm^−1^ (stretch). Formation of ester derivatives is confirmed by chemical test. (1) Place 2 mg of sample in test tube; (2) add 50 microliters of saturated hydroxylamine hydrochloride; (3) add 75 microliters of 3 molar NaOH; (4) heat, cool, then add 80 microliters 3 molar HCl; and (5) add 50 microliters of 1% FeCl_3_ which forms a red-brown mixture to confirm the presence of ester.

### 2.4. Culture of Bacterial Samples

 A known amount of drug to be tested was dissolved to make 1 mg/mL stock in sterile water with 0.1% HCl and filtered through 0.2 *μ*m syringe filter. Each drug was diluted in Luria-Bertani media with the addition of *E. coli *to obtain the desired concentration. An overnight growing plasmid-induced ampicillin-resistant *E. coli *was utilized for these tests. Growth inhibition was assayed by optical absorbance (600 nm) and determination of colony-forming units. 

The five iterations of drugs with bacteria were as follows: (1) ciprofloxacin alone; (2) mixture of ciprofloxacin (15 milligrams) plus drug 2 (34 milligrams); (3) mixture of ciprofloxacin (20 milligrams) plus drug 3 (49 milligrams); (4) mixture of ciprofloxacin (24 milligrams) plus drug 4 (63 milligrams); and (5) mixture of ciprofloxacin (33 milligrams) plus drug 2 (33 milligrams) plus drug 3 (29 milligrams) plus drug 4 (34 milligrams). These mixtures 1 from 4 to placed into tissue culture with penicillin are resistant *E. coli* at known concentrations to determine extent of antibacterial activity.

## 3. Results and Discussion

 Necrotizing soft-tissue infection is highly lethal and leaves patients, even those receiving prompt and adequate care, requiring reconstructive intervention and rehabilitation. Some studies have concluded that time leading to operative intervention is the most important determinant of mortality [2]. Similarly, other observations conclude that optimal treatment is attained with early diagnosis, radical surgical debridement of all necrotic tissue, application of broad-spectrum antibiotics, and aggressive nutritional support [12]. Administration of broad-spectrum antibiotics must be initiated at the earliest sign. Ciprofloxacin is a fluoroquinolone type of antimicrobial agent that inhibits bacterial enzyme DNA gyrase needed for replication of DNA. It is effective with both oral or intravenous administration and demonstrates potent antibacterial activity against most gram-negative bacteria and many gram-positive bacteria (having particularly good activity against gram-negative bacteria) [13]. Ciprofloxacin reaches concentrations in most tissues and body fluids sufficient to inhibit the majority of susceptible pathogens [13]. Ciprofloxacin is an effective treatment for those infections of the skin and soft-tissues [13]. Orally administered ciprofloxacin appears to be at least as effective as orally administered trimethoprim, cotrimoxazole (trimethoprim/sulfamethoxazole), amoxicillin, and amoxicillin/clavulanic acid and just as effective as parenteral agents ceftriaxone, cefamandole, ceftazidime, cefotaxime [13]. Ciprofloxacin is effective for both* Streptococcus pyogenes *and* Staphylococcus aureus* [14] and is prescribed for polymicrobial anaerobic infections that involve clostridia [15]. 

**Figure 1:**
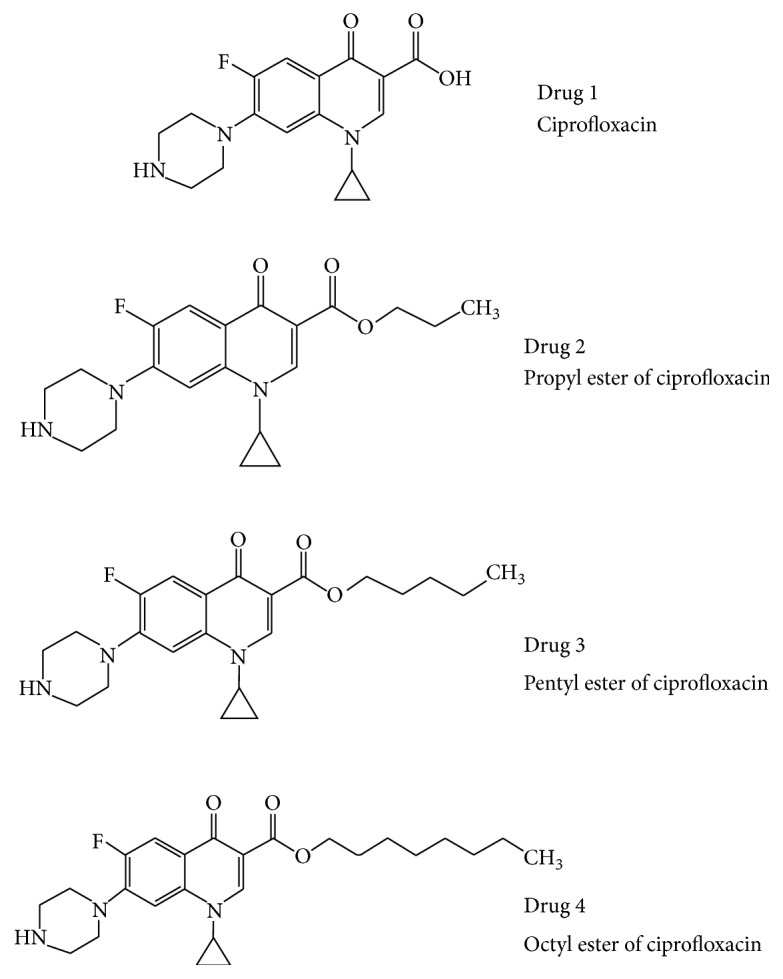
Comparative molecular structures of ciprofloxacin and ester derivatives. Drug 2 is the n-propyl derivative followed by drug 3 (n-pentyl derivative) and drug 4 (n-octyl derivative). The formation of an ester group (–C(O)OR) to replace the original carboxyl group (–C(O)OH) and the type of ester moiety imparts substantial changes in molecular properties. The alteration in molecular properties involves descriptors such as Log *P*, polar surface area, molecular weight, and molecular volume. Beneficial changes will enhance drug likeness of the medicament.

Ciprofloxacin is clearly a versatile and potent antibacterial agent that was utilized in this study along with three aliphatic ester derivatives of ciprofloxacin. The molecular structures of parent ciprofloxacin (drug 1), n-propyl ester (drug 2), n-pentyl ester (drug 3), and n-octyl ester (drug 4) are presented in Figure 1 for comparison. Note that the original carboxyl group (–C(O)OH) of the parent ciprofloxacin is replaced by ester functional groups (–C(O)OR) in subsequent derivatives drug 2, drug 3, and drug 4. All other structural features of ciprofloxacin are preserved in derivatives. 

Various molecular properties were determined for these agents with descriptors included in Table 1. All agents show zero violations of the Rule of 5 which is a favorable outcome when evaluating drug likeness [16]. The Rule of 5 serves as a guideline for screening potential drug candidates for effective absorption or permeation. In general, an orally active drug will have no more than one violation of the following criteria [16]: (1) more than 5 Hydrogen-bond donors; (2) the molecular weight over 500; (3) the Clog *P* is over 5 (or *M*Log *P* is over 4.15); and (4) The sum of nitrogen and oxygen is over 10. Subsequently ciprofloxacin and all derivatives are expected to be effective and orally active with favorable permeation and absorption. Log *P* values of these agents are very highly correlated (*r* > 0.9900) with number of atoms, molecular weight, and number of rotatable bonds. Polar surface area (PSA) is highly inversely correlated (−1.000 < *r* < −0.7000) with number of atoms, molecular weight, and molecular volume. Note that as the length of the ester functional group increases from drug 2 (n-propyl) to drug 4 (n-octyl), the number of –OH, –NH_n_, oxygens, and nitrogen remains constant (thus restraining the PSA to nominal 63.6 Angstroms^2^). As the numerical value of Log *P* increases beginning from the parent ciprofloxacin through the lengthening ester moiety, the antibacterial agents itself becomes more lipophilic (i.e., less hydrophilic). The decreasing hydrophilic property of the derivatives (e.g., the Log**** *P* values increase numerically) compared to parent ciprofloxacin premises an increased permeability through lipophilic membranes by these ester derivatives.

**Figure 2:**
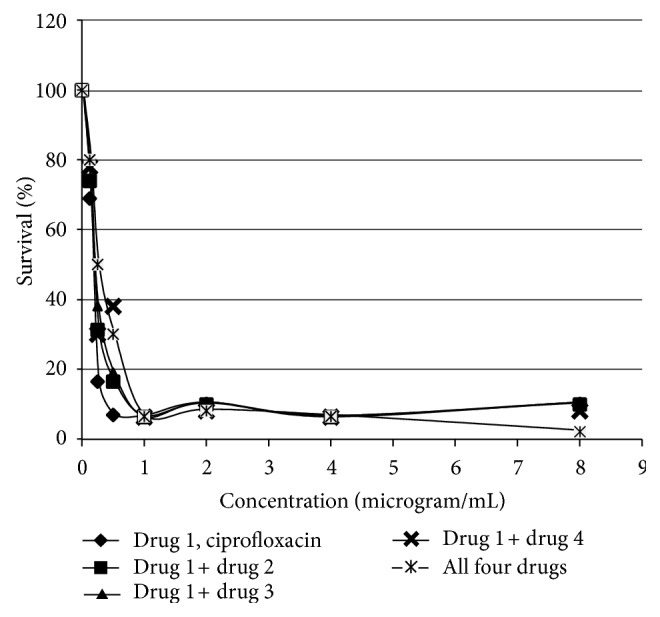
Inhibition of penicillin-resistant *Escherichia coli* by parent ciprofloxacin and combinations of parent ciprofloxacin with various ester derivatives. Note that the observed bacterial death rate is greater than 60% (e.g., percent survival less than 40%) at concentrations less than 1 microgram per milliliter for all combinations. The rate of bacterial death is greater than 80% at concentrations greater than one microgram per milliliter for all combinations. Ciprofloxacin with ester derivatives incurs bacterial death with the additional benefit of enhanced tissue penetration of the antibacterial. Drug combinations are describe previously (1), (2), (3), (4), and (5).

Growth inhibition of penicillin-resistant *E. coli* was evaluated via in vitro addition of known amounts of ciprofloxacin with/without derivatives in the presence of *E. coli.* The ester derivatives were combined with parent ciprofloxacin individually then as a mixture to demonstrate the efficacy of administering broad-spectrum antimicrobials having multiple levels of Log**** *P* (i.e., multiple levels of cell membrane penetration). The outcome of in vitro titration of drugs 1 through 4 showed extremely strong bacterial inhibition even to concentration level below one microgram per milliliter (see Figure 2). The results show that all combinations of drugs (ciprofloxacin plus drug 2 or drug 3 or drug 4) and the mixture of all four drugs (ciprofloxacin and drug 2, 3, 4) induced greater than 80% bacterial death at one microgram per milliliter concentration. The five iterations of drug + bacteria were as follows: (1) ciprofloxacin alone (more than 80% bacteria death at one microgram/milliliter); (2) ciprofloxacin plus drug 2 (more than 80% bacteria death at one microgram/milliliter); (3) ciprofloxacin plus drug 3 (more than 80% bacteria death at one microgram/milliliter); (4) ciprofloxacin plus drug 4 (more than 80% bacteria death at one microgram/milliliter); and (5) all drugs combined (more than 80% bacteria death at one microgram/milliliter). The extent of bacterial death remains greater than 80% across the concentration range of one microgram per milliliter to eight micrograms per milliliter. Figure 2 shows clearly a profound bacterial inhibition at very low concentrations for these drug combinations and a demonstration of the efficacy of multiple levels of Log *P* (e.g., cell membrane penetration) internal to this broad-spectrum antibiotic. In addition to Figure 2 the numerical values of reduced bacterial survival is presented in Table 2 as percent of survival to concentration for each of ciprofloxacin and it's combination with ester derivatives. These numerical values for decreased bacterial survival (plotted in Figure 2) indicate clearly that the combination of ciprofloxacin with various ester derivatives expresses very strong bacterial growth inhibition. In addition, the enhanced tissue penetration by derivatives of ciprofloxacin is then anticipated to improve antibacterial activity. 

Necrotizing fasciitis can be classified according to the depth of infection as well as microbial origination [2]. Necrotizing fasciitis is an infection of the deeper layers of skin, notably the hypodermis layer (referred to as superficial fascia, subcutis, and subcutaneous layer), that spreads across the fascial plane within the subcutaneous tissue [2, 3]. The dermal permeability coefficient (*K*_*p*_) is the most used and effective for predicting percutaneous penetration of drugs in quantitative structure property activity relationships and basis predictions on the physiochemical properties of the penetrant drug [17]. The physical and chemical properties of a drug (or carrier vehicle) have a decisive effect on the drugs permeation through the dermal tissues [17]. Previous studies have confirmed that *K*_*p*_ is a successful model for estimating drug proliferation based on molecular properties [18]. The rate of drug permeation in centimeter per hour is determined from the following equation (MW; molecular weight) [19]:

(1) $$\begin{array}{c} \log K_{p}\;\left(\text{cm}/\text{hr}\right)=-2.72+0.71\text{Log}\;P-0.0061\;\text{MW}. \end{array}$$

**Figure 3:**
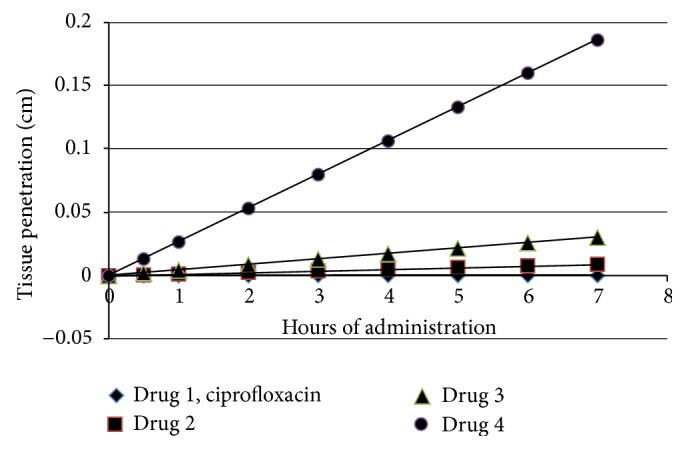
Enhanced tissue penetration over time period of 7 hours utilizing the dermal permeability coefficient (*K*_*p*_) and the dermal absorbed dose per event for each compound. Note that the depth of penetration into tissue increases per time period of 7 hours as the length of the ester moiety (aliphatic) increases. The penetration of tissue is greatly enhanced with the placement of an n-octyl ester moiety in place of the original carboxyl group of parent ciprofloxacin. The enhancement of tissue penetration by the n-propyl, n-pentyl, and vast improvement by n-octyl ester derivatives of the broad-spectrum ciprofloxacin will powerfully improve the distribution of the antibacterial medicament within infected tissue.

Values of *K*_*p*_ for drugs 1 through 4 are shown in Table 3 as rates expressed in centimeter (cm) per hour. Note that the rate of tissue permeation represented at *K*_*p*_ values increases from 2.87*E* − 05 cm/hour to as high as 26.6*E* − 03 cm/hour for drug 4 despite the increase of molecular weight of the ciprofloxacin derivatives due to lengthening of the aliphatic ester group (–C(O)OR). As values of Log *P* increase for each derivative, so does the rate of tissue penetration as represented by *K*_*p*_. The distance of tissue penetration can be determined over a period of seven hours and plotted for comparison for each ester derivative and parent ciprofloxacin (see Figure 3). Note that all ester derivatives of ciprofloxacin have greater distance of penetration than the parent ciprofloxacin. The enhanced penetration is exceedingly noticed with the n-octyl ester of ciprofloxacin having distance traveled to include 0.0266 cm at one hour and 0.106 cm at four hours. Clearly the ester derivatives enhance penetration by this broad-spectrum antibiotic.

Similarly the penetration into the central nervous system (CNS) has been studied and brought forth methods of predicting drug penetration through the blood-brain barrier (BBB). Penetration through the BBB is particularly difficult due to the complex cellular system required to maintain homeostasis of the CNS [20]. Utilizing the following equation for Log BB, where BB = [Concentration of drug in brain/concentration of drug in blood], then reckoning of the extent of BBB penetration can be accomplished (PSA, polar surface area) [20]:

(2) $$\begin{array}{c} \;\;\;\;\;\;\;\text{Log}\;\text{BB}=-0.0148\left(\text{PSA}\right)+0.152\left(\text{Log}\;P\right)+0.139. \end{array}$$

Determining Log BB and BB for all drugs (see Table 3) again shows that the tissue penetration is substantially enhanced by substituting the carboxyl group of ciprofloxacin with ester functional groups. Note that values of BB increase from 0.085 (ciprofloxacin) to as high as 0.454 for the n-octyl ester derivative of ciprofloxacin. Clearly the presence of the ester functional group within the molecular scaffolding of ciprofloxacin benefits and enhances the tissue penetration of this broad-spectrum antibiotic.

Other studies have shown patently that the rate of gram-negative multidrug resistance is aggravating and threatens the effectiveness of recent broad-spectrum antibiotics [21]. There exists a portentous need for invigoration of the process to discover new antibiotics or contend with a growing risk that infections, especially in hospitals (nosocomial), will become more difficult to treat or untreatable [22]. Three novel aliphatic ester derivatives of ciprofloxacin are shown in this study to be effective in suppressing growth of penicillin-resistant *E. coli* when introduced simultaneously with the parent drug and expressing differing levels of cell membrane solubility reflected by Log *P* values. Previous studies have shown that esters of ciprofloxacin are highly effective for inhibiting the growth of methicillin-resistant *Staphylococcus aureus* (MRSA) and methicillin susceptible *Staphylococcus aureus* (MSSA) [23]. In addition, other investigations have determined that ester derivatives of ciprofloxacin effectively inhibit ampicillin-resistant *E. coli* [24]. Other studies have shown the efficacy of pursuing new formulations and dosing agendas to contend with antimicrobial resistance [25]. In addition, many of the most recent antibiotic agents receiving government approval are in fact enhanced derivatives from already established classes of antibiotics [26].

The parent compound ciprofloxacin has a basic amino and an acidic carboxyl group (–COOH) in its structure, allowing potential zwitterion form. However, the acidic carboxyl group is lost to become ester groups in the derivative forms (retaining the amino moiety). Even these compounds in ion form find the physiological water solvent to induce more solubilization [27]. Previous studies showed that the increase in lipophilicity increased dermal and transdermal drug delivery for zwitterion drugs [28], and the electrical potential difference across the skin is too small to significantly affect permeation of ions [29]. 

Values of Log *D* are useful for evaluating drug permeation at various pH at locations of drug absorption (i.e., stomach, intestine). The coefficient Log *D* is the ratio of the sum of the concentrations of all forms of the agent (ionized plus unionized) in two phases [30]. Log *D* values for ciprofloxacin and derivatives are determined by SPARC methodology [31] and presented in Table 4 at pH 3 (stomach), pH 5.5 (intestinal absorption), and pH 7.4 (blood) [32]. It is clearly seen that at these three important locations for drug absorption the ester derivatives of ciprofloxacin have significantly increased permeation into cellular membrane compared to the parent ciprofloxacin. 

In summation, the soft-tissue infection of the superficial fascia (subcutis, hypodermis) is an infliction that may lead to the highly lethal condition referred to as necrotizing fasciitis. *E. coli* is a microbe recognized in previous studies to be associated with necrotizing fasciitis. In this study-penicillin-resistant *E. coli* was strongly inhibited with great cell death at concentrations beginning at one microgram per milliliter utilizing the broad-spectrum antibiotic ciprofloxacin in vitro with the presence of various aliphatic ester derivatives of the same. Ciprofloxacin alone as well as ciprofloxacin plus ester derivatives (n-propyl, n-pentyl, and n-octyl) in vitro culture elicited greater than 80% microbe death at one microgram per milliliter and higher. A determination of dermal permeability coefficient (*K*_*p*_) and Log BB showed clearly that Log *P*, molecular weight, and polar surface area modifications due to ester functional groups greatly benefit and enhance the tissue penetration of the ester derivatives drug 2, drug 3, and drug 4. The simultaneous presence of the parent ciprofloxacin with an ester derivative greatly inhibits the growth of penicillin-resistant *E. coli* and will enhance tissue penetration of this effective antibiotic. Also the administration of ciprofloxacin with three ester derivatives (drug 2 n-propyl; drug 3 n-pentyl; drug 4-n-octyl) induced more than 80% bacteria death at one microgram per milliliter. The administration of a broad spectrum antibiotic having duple or more Log *P* levels will enhance and benefit drug proliferation into the tissue and have a concomitant strong repression of microbes responsible for necrotizing fasciitis.

## Figures and Tables

**Table 1:** Molecular properties of drugs.

Drug	Log *P*	Polar surface area (*A* ^2^)	Number of atoms	Molecular weight	Number of oxygen and nitrogen	Number of –OH and –NH_*n*_	Violations of rule of 5	Number of rotatable bonds	Molecular volume (*A* ^3^)
Drug 1	−0.701	74.6	24	331.3	6	2	0	3	285.5
Drug 2	0.437	63.6	27	373.4	6	1	0	6	336.6
Drug 3	1.50	63.6	29	401.5	6	1	0	8	370.2
Drugs 4	3.02	63.6	32	443.6	6	1	0	11	420.6

**Table 2:** Percent survival of bacteria by dosage concentration (see Figure 2).

Concentration (microgram/mL)	Ciprofloxacin	Ciprofloxacin + Propyl Ester	Ciprofloxacin + Pentyl Ester	Ciprofloxacin + Octyl Ester	All four drugs
0	100	100	100	100	100
0.125	68.9	74	74	78	80
0.25	16.4	31.2	38	30	50
0.5	6.8	16.4	19	38	30
1	6.2	6.2	6	6.2	6.4
2	10	9.8	10.2	8	8
4	6.2	6.2	6	6.2	6.4
8	10	9.8	10.2	8	2

**Table 3:** Penetration of tissue (superficial fascia and BBB).

Drug	Log BB	BB	Dermal Permeability Coefficient *K*_*p*_ (centimeter/hour)
Drug 1, ciprofloxacin	−1.07	0.085	2.87*E* − 05
Drug 2, propyl ester	−0.735	0.184	1.29*E* − 03
Drug 3, Pentyl Ester	−0.574	0.267	4.32*E* − 03
Drug 4, octyl ester	−0.343	0.454	26.6*E* − 03

**Table 4:** Log *D* values of ciprofloxacin derivatives by pH.

pH	Ciprofloxacin	Ciprofloxacin propyl ester	Ciprofloxacin pentyl ester	Ciprofloxacin octyl ester
3.0	0.09	0.37	1.32	2.69
5.5	−0.10	0.42	1.37	2.73
7.4	−0.56	1.40	2.35	3.70

## Abbreviations

*E. coli*: *Escherichia coli*
RA: Rheumatoid arthritis
PSA: Polar surface area
LB: Luria-Bertani
MW: Molecular weight
CNS: Central nervous system
BBB: Blood-brain barrier
BB: C_brain_/C_blood_
MRSA: Methicillin resistant *Staphylococcus aureus *
MSSA: Methicillin susceptible *Staphylococcus aureus*.
